# Editorial Note: Extensive culturomics of 8 healthy samples enhances metagenomics efficiency

**DOI:** 10.1371/journal.pone.0324350

**Published:** 2025-05-13

**Authors:** The PLOS One Editors

The *PLOS One* Editors issue this notice to update the previously published Expression of Concern on this article [1,2].

Following the publication of the article and Expression of Concern [1,2], PLOS investigated concerns pertaining to the reported ethical approval and the article’s adherence to *PLOS One*’s research ethics and reporting requirements.

Specifically, the research ethics concerns included that the study involved human participants but the article did not report Comité de Protection des Personnes ethics approval, and the ethics approval number #2016–011 cited in [1] was also reported in > 50 other published articles despite apparent differences in the aims and objectives, study locations, study populations, age ranges, methodologies, types of samples collected, and types of consent described in these studies. S1 File contains a summary of articles citing ethics approval number #2016–011 of which PLOS is aware.

In addition, the *PLOS One* article [1] did not report sufficient information about participant recruitment and eligibility criteria as would be needed to replicate this study, and it did not report when the samples used in this study were collected.

A representative of the Aix-Marseille Université Ethics Committee stated that the stool samples collected in this study are considered human waste, and that the study did not require ethics approval from a Comité de Protection des Personnes according to French law. Furthermore, the representative indicated that they were not concerned about the ethics approval number reuse and stated that #2016.011 is a “generic” approval.

The ethics approval (#2016.011) was issued by the Ethics Committee of the IHU Mediterranean Infection and Institut Fédératif de Recherche 48 on 21 September 2016. The approval is for an epidemiological study of human microbiota using culturomics and metagenomics. It approves use of anonymized stool samples collected with patient consent but does not mention healthy subjects or include other study-specific details such as approved study dates, sample sizes, or a description of the participant population(s). PLOS has unresolved questions about whether the approval addressed applicable ethics requirements for this study [1,2] which involved stool samples collected from ‘apparently healthy subjects’. PLOS also remains concerned about the widespread use of the ethics approval number and noted that other articles citing approval #2016.011 report collection of sample types not listed in the approval document.

## In addition

- In response to editorial queries the authors did not clarify when the samples used in this study [1] were collected. This information is needed to evaluate the article’s compliance with the PLOS Human Subjects Research policy.
- PLOS identified potential competing interests between the committee that granted the ethics approval and one or more of the article’s authors.

In light of the unresolved issues, the Expression of Concern stands.

## Supporting information

S1 File Overview of 55 articles referencing ethics approval number #2016–011. Note that the #2016–011 approval document only indicates approval for the use of stool samples, although these articles report collection of other samples including urine, oral fluids, sputum, saliva, vaginal swabs, bronchial aspirates, dental plaque, and bronchoalveolar lavage.
(XLSX)
